# Loss of CCDC6 Affects Cell Cycle through Impaired Intra-S-Phase Checkpoint Control

**DOI:** 10.1371/journal.pone.0031007

**Published:** 2012-02-17

**Authors:** Angeliki Thanasopoulou, Dimitrios J. Stravopodis, Konstantinos S. Dimas, Juerg Schwaller, Ema Anastasiadou

**Affiliations:** 1 Department of Genetics & Gene Therapy, Biomedical Research Foundation of Academy of Athens, Athens, Greece; 2 Department of Cell Biology & Biophysics, Faculty of Biology, University of Athens, Athens, Greece; 3 Laboratory of Pharmacology, Faculty of Medicine, University of Thessaly, Larissa, Greece; 4 Department of Biomedicine, Basel University Medical School, Basel, Switzerland; Texas A&M University, United States of America

## Abstract

In most cancers harboring Ccdc6 gene rearrangements, like papillary thyroid tumors or myeloproliferative disorders, the product of the normal allele is supposed to be functionally impaired or absent. To address the consequence of the loss of CCDC6 expression, we applied lentiviral shRNA in several cell lines. Loss of CCDC6 resulted in increased cell death with clear shortening of the S phase transition of the cell cycle. Upon exposure to etoposide, the cells lacking CCDC6 did not achieve S-phase accumulation. In the absence of CCDC6 and in the presence of genotoxic stress, like etoposide treatment or UV irradiation, increased accumulation of DNA damage was observed, as indicated by a significant increase of pH2Ax Ser139. 14-3-3σ, a major cell cycle regulator, was down-regulated in CCDC6 lacking cells, regardless of genotoxic stress. Interestingly, in the absence of CCDC6, the well-known genotoxic stress-induced cytoplasmic sequestration of the S-phase checkpoint CDC25C phosphatase did not occur. These observations suggest that CCDC6 plays a key role in cell cycle control, maintenance of genomic stability and cell survival and provide a rational of how disruption of CCDC6 normal function contributes to malignancy.

## Introduction

Coiled-coil domain containing 6 (CCDC6, also known as H4/D10S170 or PTC1) encodes for a ubiquitously expressed protein, highly conserved across species, that is frequently rearranged in human malignances. It was initially isolated and characterized due to its participation in RET/PTC1 oncogene, the product of inversion inv(10)(q11.2q21) which is present in approximately 20% of papillary thyroid carcinoma (PTC) cases [1]. It also forms H4/PDGFRβ, the fusion gene product of the translocation t(5;10)(q33;q22), occurring in sporadic cases of atypical chronic myeloid leukemia [2]–[4].

The oncogenic activity of CCDC6 fusion proteins has been demonstrated *in vitro* and *in vivo* [3], [5], [6]. There is compelling evidence showing that RET/PTC rearrangements are early genetic events in the process of cancer formation [7]–[9]. The presence of RET/PTC1 in adenomas and benign tumors [10]–[12] indicates that RET/PTC1 probably acts synergistically with other factors that lead to malignancy. Interestingly, the vast majority of PTC bearing RET/PTC1 failed to express wild type CCDC6 from the non-rearranged allele, suggesting a potential tumor suppressor function of this gene [10]. In addition, in cases expressing the normal allele, CCDC6 seems to be functionally impaired through heterodimerization with the coil-coiled domain of the fusion protein [13], [14]. These data indicated that loss of normal CCDC6 might support oncogenic transformation. Moreover, normal CCDC6 might be a positive regulator of apoptotic cell death [14], [15]. Furthermore, recent work has suggested that CCDC6 might be functionally implicated in the cellular DNA damage response [15]. To study the functional consequences of loss of CCDC6 we applied a highly efficient lentiviral shRNA knock down strategy in several human cancer cell lines. We found that loss of CCDC6 resulted in distinct S-phase cell cycle defects, increased genomic instability and cell death.

## Results

### Efficient CCDC6 silencing alters proliferation rate and significantly increases cell death

**Figure 1:**
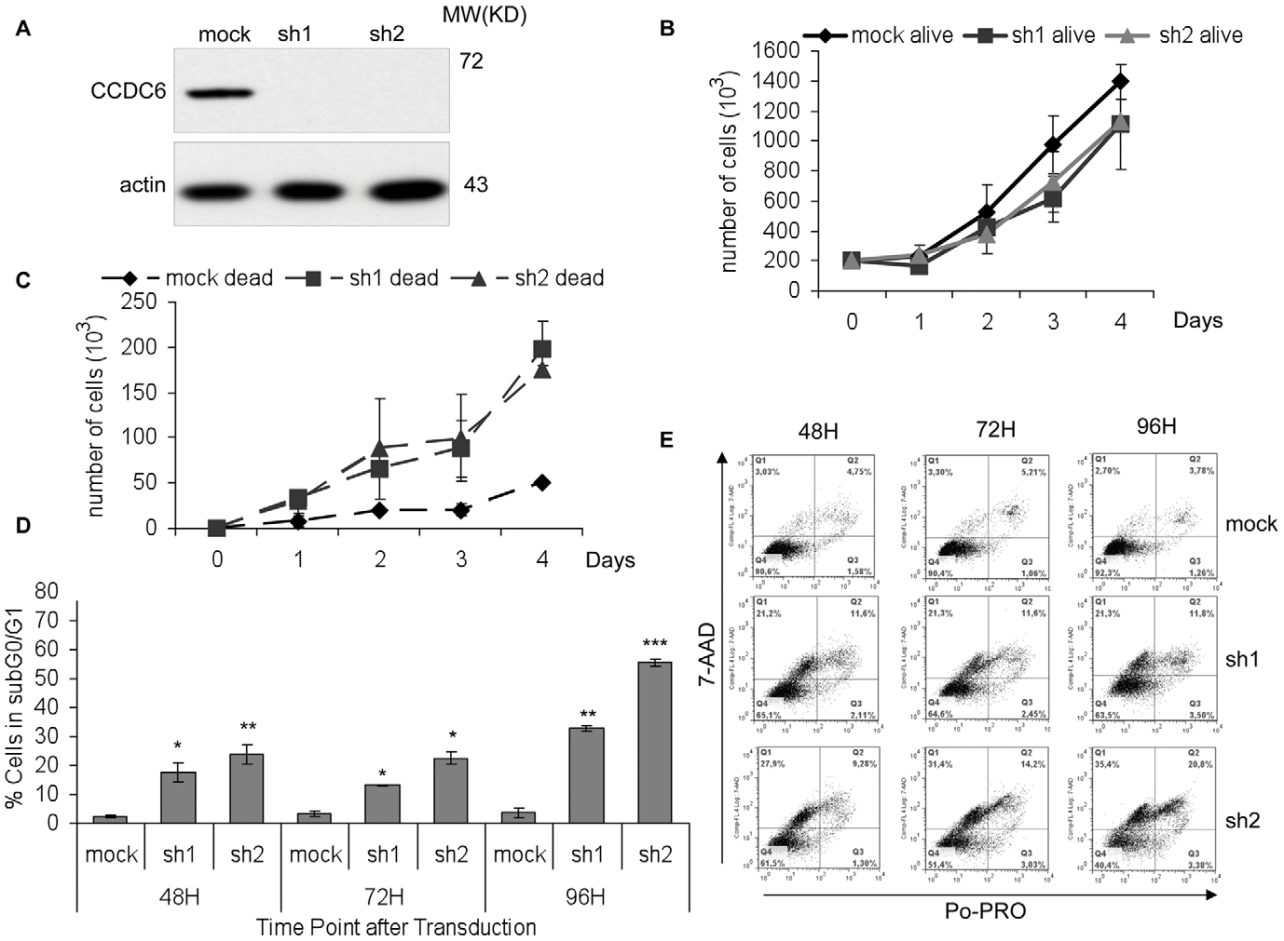
CCDC6 knock down alters proliferation rate and increases cell death *in vitro*. Cells were transduced using lentivirus, expressing two different small hairpins for CCDC6, labeled as sh1 and sh2 and cultured for 48 hours. The same viral vector was applied as control (mock: mock transduced). (**A**) Western blot analysis using anti-CCDC6 mouse monoclonal antibody demonstrated the efficient knock down of CCDC6 protein expression. Growth curves were performed in triplicates using trypan blue dye exclusion for counting the alive (**B**) and the dead cells (**C**). Decreased proliferation rate and increased cell death was observed in the absence of CCDC6. (**D**) The subG_0_/G_1_ population, as measured by flow cytometry, is indicative of apoptosis and is significantly increased following CCDC6 knock down. The percentage of survival was calculated for each time point by excluding both early apoptotic and dead cells. (**E**) Apoptotic cell death was analyzed by Po-PRO and 7-ADD staining. The Po-PRO single-positive cells are early apoptotic while the double positive stained cells for Po-PRO and 7-AAD are late apoptotic and dead cells. All assays were performed in three independent experiments.

Two lentiviruses expressing different CCDC6 shRNAs were applied to HCT116, HeLa and MCF7 cells. As show in **Figure 1A**, expression of either shRNA, resulted in highly significant reduction of the protein levels of CCDC6. The impact of CCDC6 knock down on proliferation and survival of HCT116 cells was studied by trypan blue dye exclusion and counting the number of alive and dead cells for 4 consecutive days (**Figure 1B**). A slight growth reduction of CCDC6 knock down cells was observed compared to mock transduced control cells. The reduced growth was associated with significantly increased cell death (72 hours: ρ_sh1_ = 9.7 10^−13^, ρ_sh2_ = 3.8 10^−07^ and 96 hours: ρ_sh1_ = 9.1 10^−07^, ρ_sh2_ = 2.4 10^−08^), suggesting that cells were not growth arrested but rather cycling before cell death (**Figure 1B**). To further investigate the observed cell death, we performed flow cytometry gating for the cell population in subG_0_/G_1_ with less than 2n DNA content, corresponding to apoptotic and dead cells (**Figure 1C**). This population was significantly increased (48 hours: ρ_sh1_ = 2.8 10^−4^, ρ_sh2_ = 8.19 10^−5^, 72 hours: ρ_sh1_ = 0.0318, ρ_sh2_ = 0.0259, 96 hours: ρ_sh1_ = 0.011, ρ_sh2_ = 3.9 10^−4^) upon CCDC6 knock down compared to the control cells, at all time points measured. We further analyzed apoptotic cell death by staining for Po-PRO and 7-AAD. The double positive population corresponding to late apoptosis was significantly increased (48 hours: ρ_sh1_ = 0.001853, ρ_sh2_ = 0.003125, 72 hours: ρ_sh1_ = 0.006971, ρ_sh2_ = 0.000968) in CCDC6 knock down cells, furthermore suggesting that efficient silencing of CCDC6 results in apoptotic cell death. Similar results were obtained in MCF7 and HeLa cells (data not shown). Consequently, we demonstrated that absence of CCDC6 results in increased cell death without growth arrest.

### Loss of CCDC6 expression results in aberrant S-phase cell cycle progression

**Figure 2:**
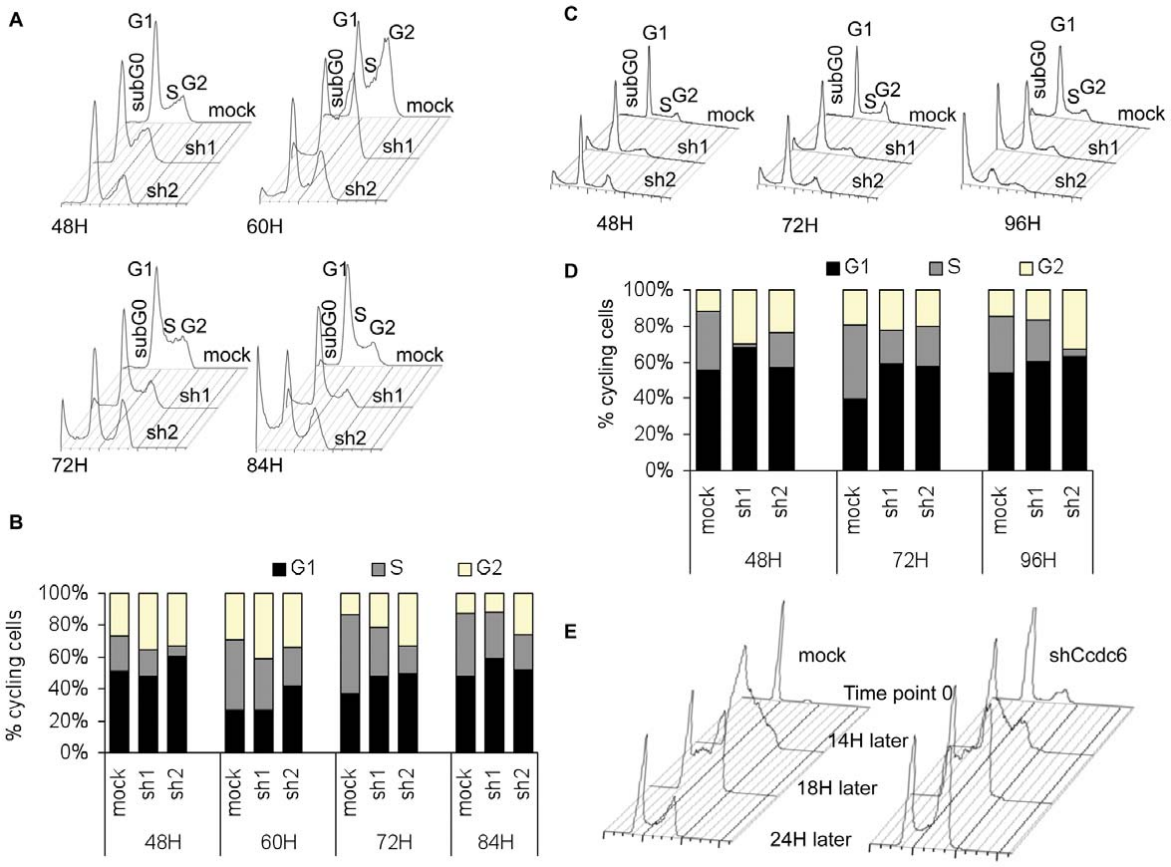
Normal Cell cycle progression is altered upon CCDC6 knock down. Cell cycle analysis was performed using propidium iodide (PI) staining and measuring the DNA content, at the indicated time points, starting 48 hours after transduction. Cell cycle was analyzed with FlowJo software and Jean-Fox algorithm. In all time points both in HCT116 (**A**), (**B**) and HeLa (**C**), (**D**) the percentage of cells in the S phase is reduced upon knock down of CCDC6 in comparison to the control (mock). One, out of three, representative experiment is shown. (**E**) HCT116 cells were synchronized by serum starvation for 48 hours followed by restimulation with 5% of FCS. CCDC6 knock down resulted in incomplete arrest at G_1_ and not total synchronization, as the control cells. 14 hours after serum stimulation the majority of the control cells are in S phase while CCDC6 knock down cells demonstrated a delay in S phase entering. 4 hours later, control and CCDC6 knock down cells showed the same profile, suggesting shorter duration of S upon CCDC6 knock down. 24 hours later, control cells were cycling normally and CCDC6 knock down cells exhibited a delay in completing G_2_ phase and re-entering G_1_.

To study the effect of CCDC6 knock down on cell cycle progression, we transduced HCT116 and HeLa cells with the shRNA expressing lentiviruses and analyzed cell cycle progression of the cells by PI staining, at 48, 60, 72 and 84 hours. As shown in **Figure 2**, alterations of all phases of the cell cycle could be observed. More specifically, we observed a consistent increase of the cell population in phases G_1_ and G_2_ and a decrease of cells in S phase of the cell cycle at all time points upon CCDC6 knock down, compared to mock-transduced cells. Similar results were observed both in HCT116 (**Figure 2A & B**) and in HeLa cells (**Figure 2C & D**).

To clarify the impact on cell cycle regulation we synchronized HCT116 cells by serum starvation for 48 hours and re-stimulated them by adding 5% FCS to the medium. Interestingly, synchronization of the mock-transduced control cells was superior to that obtained by the CCDC6 knock down cells (**Figure 2E**). More specifically, the majority of the mock-transduced cells were arrested in G_1_ phase (84%) while a very small percentage of the cells was distributed among S (6%) and G_2_ phase of the cycle (4%), as expected. In contrast, upon CCDC6 knock down, the cells were mainly in G_1_ (78%), with a respectful 22% of cells in G_2_; cells in S phase were nearly undetected (1%). Moreover, 14 hours after serum re-stimulation, the majority (≈90%) of the control cells was in the S phase whereas cells lacking CCDC6 exhibited a delay in G_1_/S transition. However, 4 hours later, both control and knock down cells demonstrated a similar profile. 24 hours after serum stimulation CCDC6 knock down cells accumulated in G_1_ and G_2_ of the cycle. At the latter time point, the control cells were cycling normally (**Figure 2E**). Taken together, these observations suggest that regulation of cell cycle is significantly affected by CCDC6 silencing, and the proper transition of cells through the S phase is disturbed.

### Loss of CCDC6 expression inhibits intra-S phase checkpoint activation and results in increased cell death

**Figure 3:**
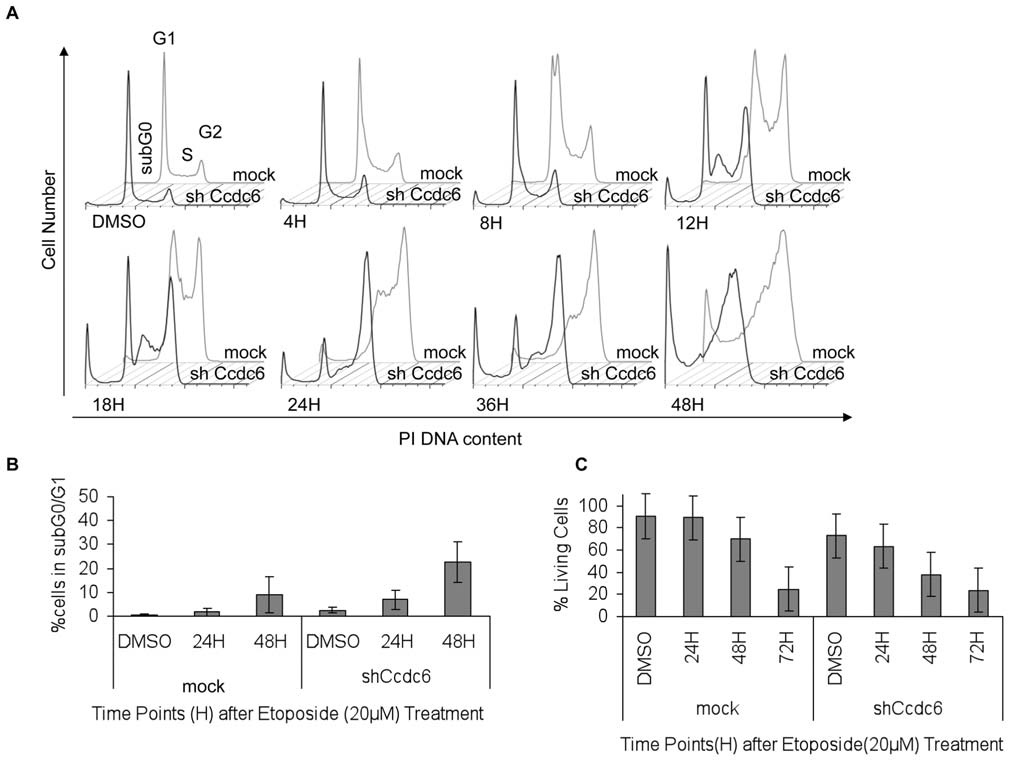
Deficient S phase checkpoint regulation upon etoposide treatment in the absence of CCDC6. (**A**) HCT116 cells were treated with 20 µM etoposide and cells were harvested at predetermined time points for cell cycle analysis. In the absence of CCDC6, no S phase accumulation is observed and the transition to G_2_ phase is accelerated. One representative experiment is shown, out of three performed. (**B**) Concomitant apoptotic cell death was quantified by measuring the subG_0_/G_1_ DNA content. CCDC6 knock down cells showed higher levels of apoptosis, at earlier time point, in comparison to the control, in response to genotoxic stress upon etoposide treatment. (**C**) The percentage of cell survival was assessed by gating for PoPRO and 7-AAD negative cells. CCDC6 knock down resulted in lower cell survival upon etoposide induced genotoxic stress. The assays were performed in triplicates.

The impact of CCDC6 on S-phase cell cycle progression was further addressed by treating mock-transduced and CCDC6 shRNA expressing HCT116 and HeLa cells with etoposide (20 µM), a well-established topoisomerase II inhibitor inducing DNA damage. Etoposide is known to activate intra-S phase checkpoint control depicted as a delay in S phase, followed by a G_2_/M arrest via activation of G_2_/M checkpoint [16]–[18]. Etoposide treatment of mock-transduced cells resulted in an initial accumulation in S phase, as early as 4 hours after etoposide addition, gradually reaching a G_2_ arrest 36 hours post treatment. In contrast, CCDC6 knock down cells showed a dramatic reduction of S phase accumulation, at all time points (**Figure 3A**). Eventually, the cells progressed and accrued in G_2_ phase, exhibiting increased cell death compared to the control. This was evidenced by the percentage of cells in subG_0_/G_1_, reaching 22.7% for knock down cells compared to 9.0% of mock transduced cells (**Figure 3A & 3B**). These results strongly suggested that the presence of CCDC6 is essential for the proper function of intra-S phase checkpoint control.

To determine the effect of CCDC6 silencing on cell survival upon etoposide-mediated genotoxic stress, the apoptotic cells were determined by Po-PRO and 7-AAD double stains. As depicted in **Figure 3C**, CCDC6 knock down resulted in a significant decrease of cell survival at 24 hours post etoposide treatment whereas the survival of control cells remained unaffected upon etoposide exposure. The difference between control and CCDC6 knock down cells remained significant at 48 hours, while at 72 hours it was eliminated, as expected, due to the etoposide toxicity. As cells lacking proper S phase checkpoint regulation, accumulate DNA damage and eventually die, these observations support the necessity of CCDC6 for the proper function of the intra-S phase checkpoint control.

### CCDC6 knockdown increases susceptibility to UV-induced DNA damage

**Figure 4:**
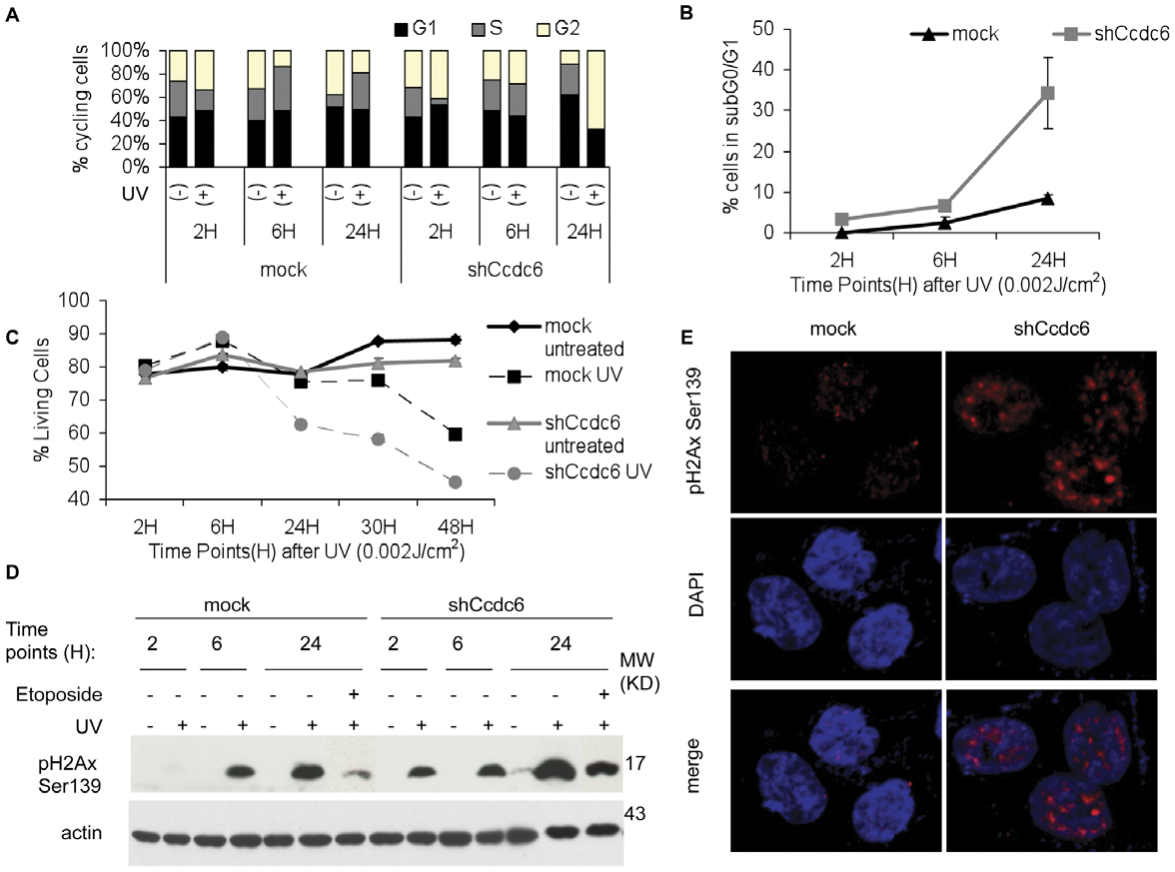
Knock down of CCDC6 increases vulnerability to genotoxic stress by UV induced DNA damage. HCT116 cells were transduced with CCDC6 shRNA expressing lentivirus or empty control followed by UV irradiation (0.002 J/cm^2^), 48 hours after transduction. Cells were harvested at 2, 6 and 24 hours after irradiation and analyzed for the cell cycle using flow cytometry. The percentages of the cell populations in each phase of the cell cycle for every time point are depicted as bars in the diagram (**A**). In control cells, UV irradiation results in G_1_ and S phase increase while in CCDC6 knock down cells UV irradiation causes a reduction of cell population in S phase and an increase in G_2_ phase. A representative experiment is shown. (**B**) The apoptotic levels were measured by flow cytometric assessment of the subG_0_/G_1_ population. Knock down of CCDC6 increase UV-mediated cell death. (**C**) Cell survival analysis by Po-PRO and 7-ADD staining (excluding the double positive cells) revealed a significantly decreased cell survival of cells lacking CCDC6. Error Bars represent 3 independent experiments. (**D**) Cell lysates of HCT116 cells treated with etoposide (20 µM) or radiated with UV (0.002 J/cm^2^) for 2, 6 or 24 hours and the untreated controls were resolved on a SDS-PAGE and probed for pH2Ax Ser139. Upon UV irradiation, pH2Ax Ser139 levels arise earlier and to a higher extent in CCDC6 knock down cells compared to the control. UV irradiation is causing high levels of pH2Ax Ser139 even in 2 hours after irradiation in CCDC6 knock down cells. The effect is similar upon etoposide treatment, although less dramatic. (**E**) Increased basal levels and nuclear foci of pH2Ax Ser139 are present in CCDC6 knock down cells, even in the absence of any additional treatment.

To further investigate the contribution of CCDC6 to intra-S phase cell cycle checkpoint, we applied genotoxic stress using UV irradiation. Previous studies have shown that UV exposure leads to the activation of an intra-S phase checkpoint that senses double strand brakes (DSB) and triggers a signaling cascade of cellular repair. The expected impact on the cell cycle profile is a delay of completion of S phase leading to transient accumulation in S phase. DNA damage beyond repair capacity results in G_2_ arrest, cell death or senescence [19]; [20]. In this context, we treated HCT116 and HeLa cells with UV-C in a DSB inducing dosage 0,002 J/cm^2^ and followed cell cycle progression with and without CCDC6 knock down. As expected, UV exposure of mock-transduced cells resulted in an increased population of cells in the G_1_ and S phase (**Figure 4A**). In contrast, upon shRNA-mediated CCDC6 knock down the cells did not accumulate in G_1_/S phase but continued cycling and entered G_2_ prematurely, suggesting that the UV-induced intra-S-phase checkpoint was impaired.

Moreover, CCDC6 knock down cells were more susceptible to UV-induced cell death compared to the controls, as determined by calculating the subG_0_/G_1_ population after PI staining. ≈35% of the knock down cells recorded in subG_0_/G_1_ while the control cells remained below 10% (**Figure 4B**), resulting in impaired cell survival (**Figure 4C**) reaching 60% from the initial 80%, 24 hours after treatment. In the latter time point, cell survival of control cells was unaffected. To follow DSBs induced by either UV irradiation or etoposide treatment, we also determined the expression of pH2Ax Ser139, an established marker for DSBs in the genome [21]. Loss of CCDC6 expression resulted in increased pH2Ax Ser139 levels upon irradiation with abundant expression as early as 2 hours post UV treatment. At the same time point, pH2Ax Ser139 levels remained undetectable in mock-transduced control cells. Overall, in all time points analyzed, the pH2Ax Ser139 protein levels were higher in the absence of CCDC6 expression (**Figure 4D**). Likewise, upon etoposide treatment, the pH2Ax Ser139 protein levels were elevated in the absence of CCDC6 (**Figure 4D**). Additionally, we visualized pH2Ax Ser139 expression by confocal microscopy and demonstrated that the levels of pH2Ax Ser139 were elevated upon knock down of CCDC6 expression in accordance to the Western blot results. Moreover, the nuclear configuration of this protein changed in the absence of CCDC6, pH2Ax Ser139 forming distinct foci. These observations furthermore suggest that CCDC6 knock down increases susceptibility to DNA damage upon genotoxic stress.

### CCDC6 knock down affects the 14-3-3σ and CDC25C regulators of G_2_/M transition

The above results demonstrate that CCDC6 contributes to the activation of the S phase checkpoints. Due to its absence there is acceleration to S phase and premature entrance to G_2_/M. The transition to G_2_/M during normal cell cycle is regulated by the activation of CDC25C. Previous studies have shown that, inactive Ser216-phosphorylated CDC25C is sequestered to the cytoplasm by the 14-3-3 proteins. Once mitosis is activated, cytoplasmic sequestration of CDC25C is inhibited by a CDC2-mediated phosphorylation of CDC25C at Ser214, leading to disassociation of 14-3-3σ and CDC25C [22]–[25].

**Figure 5:**
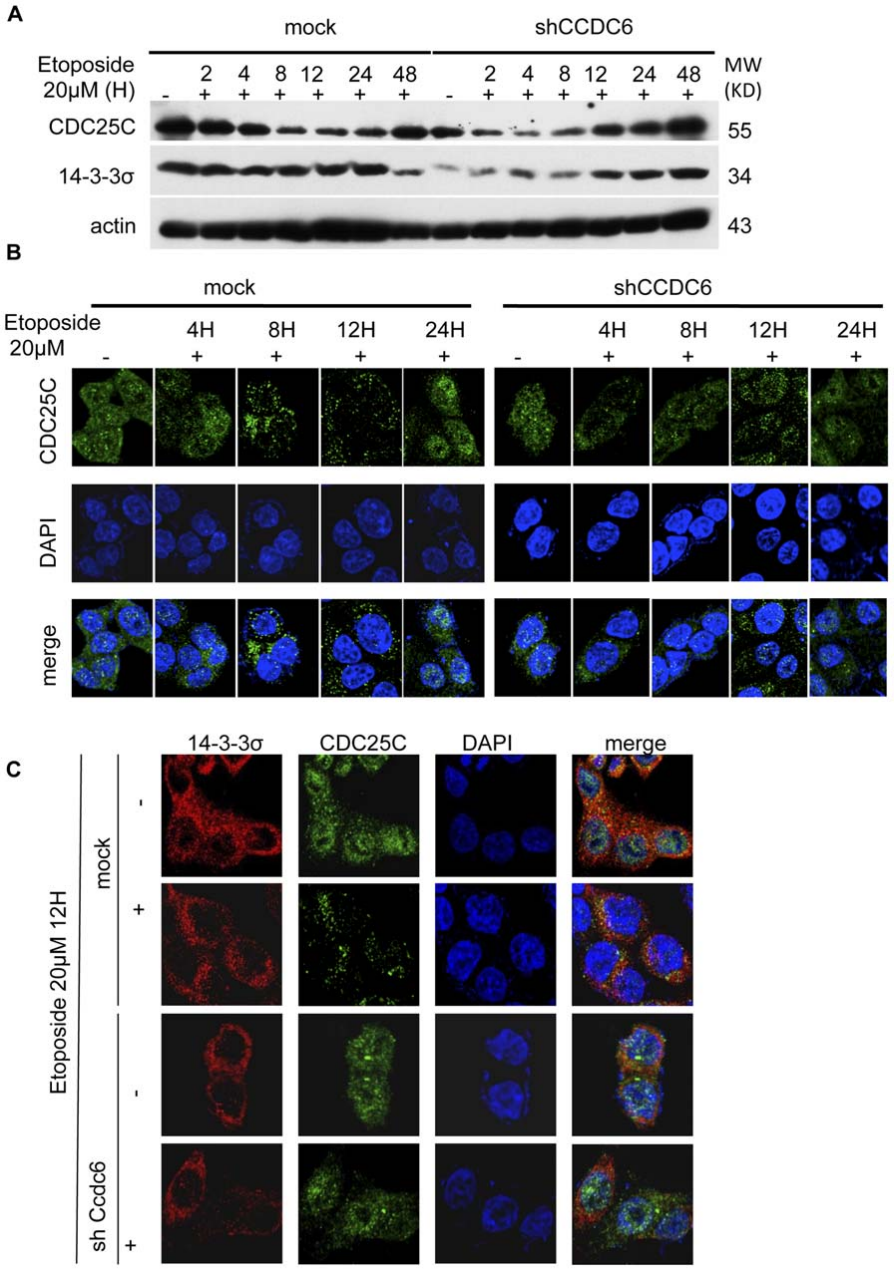
CCDC6 knock down results in altered cellular localization of CDC25C and accelerated G_2_/S transition upon etoposide-mediated genotoxic stress. Control and CCDC6 knock down HCT116 cells were treated with etoposide (20 µM). (**A**) Cell lysates of HCT116 cells treated with etoposide (20 µM) for 2, 4, 8, 12 and 24 hours and mock control treated with DMSO vehicle were resolved on a SDS-PAGE and probed for 14-3-3σ and CDC25C. 14-3-3σ protein levels were down-regulated in the absence of CCDC6 protein expression and the CDC25C protein level regulation was altered. (**B**) Cells grown on cover slips were exposed to etoposide for 4, 8, 12, 24 hours, fixed and stained for CDC25C. In mock cells, CDC25C is kept in the cytosol upon etoposide treatment at 8 and 12 hours but is localized in the nucleus in the absence of CCDC6. (**C**) Cells exposed to etoposide for 12 hours were co-stained for CDC25C and 14-3-3σ. CDC25C is kept in the cytosol upon etoposide treatment and exhibits co-localization with 14-3-3σ (seen in yellow) but enters the nucleus in the absence of CCDC6.

We followed these S-phase checkpoint mediators, CDC25C and 14-3-3σ, upon exposure of HCT116 cells to etoposide (20 µM), in the presence or absence of CCDC6 using Western blotting and confocal microscopy. As shown in **Figure 5A**, CDC25C is steadily increased in a course of time, upon etoposide treatment. However, CCDC6 knock down resulted in an earlier increase of the CDC25C protein levels compared to mock-transduced cells. Moreover, in CCDC6 knockdown the protein levels of 14-3-3σ decreased and then increased upon etoposide treatment. In contrast, in mock-transduced cells the 14-3-3σ levels remained unchanged for 24 hours. More interestingly, absence of CCDC6 altered the localization of these molecules (**Figures 5B & C**). More specifically, we followed the subcellular localization of CDC25C upon etoposide treatment at different time points (0, 4, 8, 12, 24, 48 hours). Cytosolic sequestration of CDC25C in cells lacking CCDC6 was not obvious in any of the time points checked whereas mock-transduced cells exhibit mainly cytosolic localization of CDC25C between 8 and 12 hours upon etoposide treatment (**Figure 5B**). In further detail, as shown in **Figure 5C**, in mock-transduced cells, CDC25C cytoplasmic localization was accompanied with significant interactions with 14-3-3σ as indicated by the numerous sites of co-localization (seen in yellow). Upon CCDC6 knockdown 14-3-3σ signals were cytosolic and down regulated and CDC25C main localization after etoposide treatment (12 hours) was found in the nucleus without any signs of co-localization with 14-3-3σ observed.

## Discussion

Several lines of evidence suggested that CCDC6-related malignancies are developed upon loss of CCDC6 normal function. First, the majority (>90%) of tumors bearing CCDC6 fusion genes do not express CCDC6 from the normal allele [10]. In addition, in the presence of the chimeric molecule, endogenous expressed normal CCDC6 protein seems to heterodimerize with the fusion product, suggesting a dominant negative effect of the fusion on CCDC6 normal function [13], [26]. Moreover, several public databases reported that a significant number of head and neck cancers exhibit considerable down-regulation of CCDC6 (www.oncomine.org, www.proteinatlas.org). Under this perspective, we have silenced expression of CCDC6 in several cell lines using a highly efficient shRNA knockdown strategy. Loss of CCDC6 resulted in apoptotic cell death with only a minor reduction in proliferation. We also reported that CCDC6 is important for normal cell cycle progression and proper function of checkpoint controls. In the absence of CCDC6, intra S phase control was deficient and this effect was enhanced upon DNA damage induced by etoposide or UV-irradiation.

A key regulator of the S phase duration and the transition to G2 is 14-3-3σ and its complexes to several partner proteins. 14-3-3-ligand association triggers a phosphorylation cascade resulting in cytoplasmic sequestration of the CDC25-B, CDC25-C phosphatases. Due to their location, the latter are kept inactive and unable to reach their targets, Cdk1/cyclin B1 and MPF complexes [27]; [28]. Upon loss of CCDC6, we observed downregulation of 14-3-3σ and altered regulation and localization of CDC25C. In fact, CDC25C enters the nucleus, were it triggers mitosis by activation of the Cdk1/cyclin B complex. These observations suggest that CCDC6 is implicated in the regulation of normal both cell cycle progression and DNA repair mechanisms most probably through co-operation with 14-3-3σ. Interestingly, a recent proteomics study proposed direct physical interaction of CCDC6 with 14-3-3σ in a PI3K kinase specific manner [29].

Cell cycle checkpoints are implemented to safeguard the genome from accumulation of genetic errors. S (synthesis) phase is undoubtedly the most vulnerable period of the cell cycle division [30] and its checkpoints are the most significant for preventing genetic instability [31]. Extensive literature shows that malfunctioned S phase and thus aberrant DNA replication can lead to increased mutagenesis and also cell death in mitosis [32]. The fact that CCDC6 is highly implicated in chromosomal rearrangements could be an additional link to its role in genomic stability and supports our findings concerning S phase deficiency upon loss of CCDC6 [33]. The spatial contiguity between CCDC6 and RET and also their location at DNA fragile sites have been accused for the high frequency of RET/PTC1 rearrangements [34]–[36]. Our data suggest, that loss of CCDC6 function can create a prosperous environment for the formation of cancer [15], [26], [37], [38]. Cells depleted for CCDC6 show increased cell death in presence or absence of genotoxic stress. These cells do not arrest in G1 or S phase upon DNA damage as expected. They proceed with DNA synthesis resulting in accumulation of DNA damage, as demonstrated by an increase of pH2Ax S139 protein levels. Our results are in agreement with previous work showing that CCDC6 down-regulation and treatment with γ-radiation resulted in increased BrdU incorporation and staining for the pH3 mitosis marker [15]. However, increased genotoxic stress-mediated DNA damage and cell death upon CCDC6 knock down cells has not been observed in the latter study. This discrepancy might be due to the higher knock down efficiency in the here presented experiments where the protective effects of CCDC6 were abolished.

Interestingly, similar cell cycle effects phenocopying CCDC6 knock down have been previously reported in leukemic cells expressing the BCR/ABL tryrosine kinase fusion oncoprotein [39], [40]. In fact expression of BCR/ABL seems also to increase DNA double-strand damage after etoposide treatment and lead to a defect in an intra-S phase checkpoint. Therefore, it will be interesting to study whether expression of the PTC1 oncogenic tyrosine kinase results in similar cell cycle checkpoint defects. Therefore, additional studies should be performed to address whether loss of normal CCDC6 might be a more common principle for other constitutively active oncogenic kinase fusions beyond the CCDC6 fusions to RET or PDGFR.

Taken together, our work suggests that loss of CCDC6 results in S phase deregulation that impairs the ability of the cell to maintain genomic integrity and creates a prosperous ground for genomic instability [30], [41]–[43]. Further specification of the exact pathways in which CCDC6 is implicated and identification of its interacting partners will be necessary to unravel the molecular mechanisms of cancers, harboring CCDC6 alterations.

## Materials and Methods

### Cell culture and treatments

Different cell lines, such as HCT116, HeLa, MCF7 and 293T were cultured as a monolayer in Dulbecco's Minimal Essential Medium (DMEM) supplemented with 10% Fetal Calf Serum, 1%P/S and 1% L-Glutamine (Invitrogen, Paislay, UK). The cells were incubated at 37°C and 5% CO_2_.

Synchronisation of HCT116s was achieved through serum deprivation for 48 hours. The cells were plated on a 6 well plate (3×10^5^ cells/well) and stimulated with DMEM supplemented with 5% of FCS. They were harvested at the indicated time points and the cell cycle was analysed.

Cells in culture were UV irradiated at 0.002 J/cm^2^ using a 254 nm lamp of a UV Stratalinker 2400 (Stratagene/Agilent Technologies, Santa Clara, CA, USA).

Cells were treated with 20 µM etoposide (Sigma/Aldrich, St. Louis, MO, USA) for the selected time points and cell cycle and apoptotic assays were performed.

### Lentiviral shRNA constructs, viral production and cell transduction

Silencing of Ccdc6 was accomplished using commercially available shRNA lentiviral based constructs (TRCN 0000083831 and TRCN0000083828) from Sigma (Sigma/Aldrich, St. Louis, MO, USA). The empty lentiviral vector TRC1 (Sigma/Aldrich, St. Louis, MO, USA) was applied as a control.

For the lentiviral supernatant production, HEK-293T cells maintained in DMEM supplemented with 5% FCS without antibiotics, on 10 cm culture plates to ∼60% confluency. Sixteen hours later transfection of HEK-293T cells took place. As a transfection reagent Lipofectamine (Invitrogen, Carlsbad, CA, USA) was used according to the manufacture's protocol. 3 µg of the envelop plasmid *pMD2G*, 3 µg of the packaging plasmid *pMDLpRRE*, 2.5 µg of the Rev-expression plasmid *pRSV/Rev* (Addgene, Cambridge, MA, USA) and 10 µg of the shRNA lentiviral construct were added in 1 mL of DMEM medium, serum and antibiotics free. 40 µL of lipofectamine was diluted in 1 mL of DMEM without serum and antibiotics and incubated for 5 min. DNA and lipofectamine parts were mixed and incubated for 20 min before added to the HEK293T cells. 24 hours later, the medium was discarded and replaced with 5 mL of DMEM with 10% FCS, without antibiotics. 24 hours later the medium was collected into a 15 mL tube and 5 mL of medium were added to the plate and the same step was repeated at 48 hours. The lentivirus-containing supernatant was filtered through a 20 µM pore filter, aliquoted to 1 mL cryotubes and quickly frozen down in liquid nitrogen. Virus was stored at −80°C.

HCT116s, HeLas and MCF7s were plated in 6 well plates (5 10^5^ cells/well) and 16 hours later were viral transduced. This was performed using 1 mL of lentiviral supernatant, diluted in 1 mL of DMEM complete medium and 4 µL of protein sulphate. Cells were centrifuged at 2,500 rpm (Heraeus Biofuge stratos centrifuge) for 90 min at RT. The medium was replaced with fresh complete DMEM and changed again 24 hours later.

### Fixation and immunostaining

Cells were cultured on cover slips, coated with polylysine. Fixation was performed in 4% paraformaldehyde (Sigma/Aldrich, St. Louis, MO, USA) solution in PBS, incubated for 10 min at RT. The excess paraformaldehyde was discarded after washing the cells three times with PBS. Permeabilisation was achieved through incubation in 0.1% Triton–X 100 solution for 10 min and then in 0.5% Triton-X 100 (Sigma/Aldrich, St. Louis, MO, USA) for 30 min. The fixed cells were incubated in 5% BSA blocking solution for 1 hour. Incubation was performed for 16 hours at 4°C in a solution containing 0.3% Triton-.X100, 0.5% BSA and the indicated primary antibody in the appropriate dilution. After washing three times with 0.3% Triton-X in PBS, cells were incubated with the secondary antibody diluted in 0.5% Triton-X in PBS and 0.5% BSA for 2 hours. The cells were washed on the cover slips and put on a slide by adding on top a droplet of clearnoutmounting medium (Invitrogen, Carlsbad, CA, USA). The secondary antibodies used were anti-goat FITCH from ZYMED, anti-mouse Alexa 555, anti-rabbit Alexa 488 from Invitrogen (Invitrogen, Carlsbad, CA, USA).

### Flow cytometric analysis

Cell death and apoptosis was measured by Membrane Permeability/Dead Cell Apoptosis Kit with Po-PRO-1 and 7-AAD Apoptosis for Flow Cytometry (Invitrogen, Carlsbad, CA, USA) as recommended by the manufacturer, using a DAKO CYAN flow cytometer (DAKO, Fort Collins, CO, USA). Cell cycle profiling was carried out by Propidium Iodide staining and analysed by FlowJo Software. The quantification of apoptosis was performed via analysis of the subG_0_. Cells were fixed with 70% ice cold ethanol and kept at −20°C over night. Then, cells were washed twice with PBS and resuspended in PI solution containing 1 mg/mL propidium iodide, 100 µg/mL RNase A, 0.001% Triton-X 100 in PBS at a concentration of 10^6^ cells/mL, incubated for 45 min at 37°C in the dark and analysed using a Dako CYAN FACS machine. The propidum iodide fluorescence emission signal was detected in the FL3 channel. At least 20,000 events were acquired.

### Confocal microscopy

Confocal fluorescence images were obtained by a Zeiss LSM710 (Zeiss, Thornwood, NY microscope) with a 63× objective. Images were analysed by Zeiss software.

### Antibodies and Western Blot analysis

Cells were harvested and lysed in RIPA (200 mM NaCl, 10 mM Tris-Hcl at pH 7.5, 0.1% SDS, 1%Triton X-100) and complete protease and phosphatase inhibitor cocktail tablets (Hoffmann-La Roche NJ, USA). The protein concentration was determined by a Bradford assay (BIORAD). Equal amounts of proteins were electrophorated and separated by 12% SDS-PAGE gels, followed by transfer onto nitrocellulose membranes. Immunoblotting was performed using various antibodies. Blotted proteins were visualized with the aid of an enhanced chemiluminescence kit (Amersham, Piscataway, NJ, USA) and imaged with Kodak Biomax Light Films (Sigma/Aldrich, St. Louis, MO, USA).

Antibodies were as follows: Anti-Ccdc6 (ab-56353), 14-3-3σ (ab1423) from Abcam (Abcam, Cambridge, UK), anti Cdc25C (sc-327), anti-actin (sc-1615) and anti pH2Ax Ser139 (07-164, Upstate) from Cell Signaling (Beverly, MA, USA). The secondary antibodies were: anti-mouse, anti rabbit and anti-goat HRP conjugated from ThermoScientific.

### Statistic analysis

Inter-group comparison was performed using a paired two sample t-test. The minimum level of statistical significance was set at a¼0.05.

## References

[1] Kondo T, Ezzat S, Asa SL (2006). Pathogenetic mechanisms in thyroid follicular-cell neoplasia.. Nat Rev Cancer.

[2] Kulkarni S, Heath C, Parker S, Chase A, Iqbal S (2000). Fusion of H4/D10S170 to the platelet-derived growth factor receptor beta in BCR-ABL-negative myeloproliferative disorders with a t(5;10)(q33;q21).. Cancer Res.

[3] Schwaller J, Anastasiadou E, Cain D, Kutok J, Wojiski S (2001). H4(D10S170), a gene frequently rearranged in papillary thyroid carcinoma, is fused to the platelet-derived growth factor receptor beta gene in atypical chronic myeloid leukemia with t(5;10)(q33;q22).. Blood.

[4] Drechsler M, Hildebrandt B, Kundgen A, Germing U, Royer-Pokora B (2007). Fusion of H4/D10S170 to PDGFRbeta in a patient with chronic myelomonocytic leukemia and long-term responsiveness to imatinib.. Ann Hematol.

[5] Santoro M, Chiappetta G, Cerrato A, Salvatore D, Zhang L (1996). Development of thyroid papillary carcinomas secondary to tissue-specific expression of the RET/PTC1 oncogene in transgenic mice.. Oncogene.

[6] Salvatore D, Barone MV, Salvatore G, Melillo RM, Chiappetta G (2000). Tyrosines 1015 and 1062 are in vivo autophosphorylation sites in ret and ret-derived oncoproteins.. J Clin Endocrinol Metab.

[7] Mizuno T, Iwamoto KS, Kyoizumi S, Nagamura H, Shinohara T (2000). Preferential induction of RET/PTC1 rearrangement by X-ray irradiation.. Oncogene.

[8] Nikiforov YE (2002). RET/PTC rearrangement in thyroid tumors.. Endocr Pathol.

[9] Viglietto G, Chiappetta G, Martinez-Tello FJ, Fukunaga FH, Tallini G (1995). RET/PTC oncogene activation is an early event in thyroid carcinogenesis.. Oncogene.

[10] Sheils OM, O'Leary JJ, Sweeney EC (2000). Assessment of ret/PTC-1 rearrangements in neoplastic thyroid tissue using TaqMan RT-PCR.. J Pathol.

[11] Elisei R, Romei C, Vorontsova T, Cosci B, Veremeychik V (2001). RET/PTC rearrangements in thyroid nodules: studies in irradiated and not irradiated, malignant and benign thyroid lesions in children and adults.. J Clin Endocrinol Metab.

[12] Guerra A, Sapio MR, Marotta V, Campanile E, Moretti MI (2011). Prevalence of RET/PTC rearrangement in benign and malignant thyroid nodules and its clinical application.. Endocr J.

[13] Tong Q, Li Y, Smanik PA, Fithian LJ, Xing S (1995). Characterization of the promoter region and oligomerization domain of H4 (D10S170), a gene frequently rearranged with the ret proto-oncogene.. Oncogene.

[14] Celetti A, Cerrato A, Merolla F, Vitagliano D, Vecchio G (2004). H4(D10S170), a gene frequently rearranged with RET in papillary thyroid carcinomas: functional characterization.. Oncogene.

[15] Merolla F, Pentimalli F, Pacelli R, Vecchio G, Fusco A (2007). Involvement of H4(D10S170) protein in ATM-dependent response to DNA damage.. Oncogene.

[16] Nitiss JL (2009). Targeting DNA topoisomerase II in cancer chemotherapy.. Nat Rev Cancer.

[17] Kaufmann WK (1998). Human topoisomerase II function, tyrosine phosphorylation and cell cycle checkpoints.. Proc Soc Exp Biol Med.

[18] Bartek J, Lukas J (2001). Mammalian G1- and S-phase checkpoints in response to DNA damage.. Curr Opin Cell Biol.

[19] Sharpless NE, DePinho RA (2005). Cancer: crime and punishment.. Nature.

[20] Rodier F, Coppe JP, Patil CK, Hoeijmakers WA, Munoz DP (2009). Persistent DNA damage signalling triggers senescence-associated inflammatory cytokine secretion.. Nat Cell Biol.

[21] Mah LJ, El-Osta A, Karagiannis TC (2010). gammaH2AX: a sensitive molecular marker of DNA damage and repair.. Leukemia.

[22] Chan TA, Hermeking H, Lengauer C, Kinzler KW, Vogelstein B (1999). 14-3-3Sigma is required to prevent mitotic catastrophe after DNA damage.. Nature.

[23] Hermeking H, Benzinger A (2006). 14-3-3 proteins in cell cycle regulation.. Semin Cancer Biol.

[24] Hermeking H (2003). The 14-3-3 cancer connection.. Nat Rev Cancer.

[25] Boutros R, Lobjois V, Ducommun B (2007). CDC25 phosphatases in cancer cells: key players? Good targets?. Nat Rev Cancer.

[26] Grieco M, Cerrato A, Santoro M, Fusco A, Melillo RM (1994). Cloning and characterization of H4 (D10S170), a gene involved in RET rearrangements in vivo.. Oncogene.

[27] Bulavin DV, Higashimoto Y, Demidenko ZN, Meek S, Graves P (2003). Dual phosphorylation controls Cdc25 phosphatases and mitotic entry.. Nat Cell Biol.

[28] Takizawa CG, Morgan DO (2000). Control of mitosis by changes in the subcellular location of cyclin-B1-Cdk1 and Cdc25C.. Curr Opin Cell Biol.

[29] Dubois F, Vandermoere F, Gernez A, Murphy J, Toth R (2009). Differential 14-3-3 affinity capture reveals new downstream targets of phosphatidylinositol 3-kinase signaling.. Mol Cell Proteomics.

[30] Bartek J, Lukas C, Lukas J (2004). Checking on DNA damage in S phase.. Nat Rev Mol Cell Biol.

[31] Myung K, Chen C, Kolodner RD (2001). Multiple pathways cooperate in the suppression of genome instability in Saccharomyces cerevisiae.. Nature.

[32] Lau E, Jiang W (2006). Is there a pre-RC checkpoint that cancer cells lack?. Cell Cycle.

[33] Penserga ET, Skorski T (2007). Fusion tyrosine kinases: a result and cause of genomic instability.. Oncogene.

[34] Nikiforova MN, Stringer JR, Blough R, Medvedovic M, Fagin JA (2000). Proximity of chromosomal loci that participate in radiation-induced rearrangements in human cells.. Science.

[35] Savage JR (2000). Cancer. Proximity matters.. Science.

[36] Gandhi M, Dillon LW, Pramanik S, Nikiforov YE, Wang YH (2010). DNA breaks at fragile sites generate oncogenic RET/PTC rearrangements in human thyroid cells.. Oncogene.

[37] Leone V, Mansueto G, Pierantoni GM, Tornincasa M, Merolla F (2010). CCDC6 represses CREB1 activity by recruiting histone deacetylase 1 and protein phosphatase 1.. Oncogene.

[38] Puxeddu E, Zhao G, Stringer JR, Medvedovic M, Moretti S (2005). Characterization of novel non-clonal intrachromosomal rearrangements between the H4 and PTEN genes (H4/PTEN) in human thyroid cell lines and papillary thyroid cancer specimens.. Mutat Res.

[39] Dierov J, Dierova R, Carroll M (2004). BCR/ABL translocates to the nucleus and disrupts an ATR-dependent intra-S phase checkpoint.. Cancer Cell.

[40] Dierov J, Sanchez PV, Burke BA, Padilla-Nash H, Putt ME (2009). BCR/ABL induces chromosomal instability after genotoxic stress and alters the cell death threshold.. Leukemia.

[41] Shen Z (2011). Genomic instability and cancer: an introduction.. J Mol Cell Biol.

[42] Shimada M, Nakanishi M (2006). DNA damage checkpoints and cancer.. J Mol Histol.

[43] Motoyama N, Naka K (2004). DNA damage tumor suppressor genes and genomic instability.. Curr Opin Genet Dev.

